# Repellent effect of local indigenous knowledge-based repellent in Nakhon Si Thammarat, Thailand, against *Aedes aegypti* mosquito

**DOI:** 10.1016/j.heliyon.2023.e21589

**Published:** 2023-10-31

**Authors:** Danita Champakaew, Palita Rattanasophon, Chayanid Phannasee, Wanwisa Saehao, Chutipong Sukkanon, Jitrawadee Intirach, Anuluck Junkum, Benjawan Pitasawat

**Affiliations:** a School of Public Health, Walailak University, Thailand and Excellent Center for Dengue and Community Public Health (EC for DACH), 80160, Nakhon Si Thammarat, Thailand; b Public Health Program in Community Public Health, School of Public Health, Walailak University, Nakhon Si Thammarat, 80160, Thailand; c School of Allied Health Sciences, Walailak University, Nakhon Si Thammarat, 80160, Thailand; d Hainan General Hospital, Hainan Affiliated Hospital of Hainan Medical University, Haikou 570100, China; e Department of Parasitology, Faculty of Medicine, Chiang Mai University, Chiang Mai, 50200, Thailand

## Abstract

Dengue fever has been a significant disease in Thailand for a long time, ranking it as one of the major health problems in the country. Management of the adult stage of mosquito vectors is approached by applying various synthetic chemicals such as adulticides, attractants, deterrents, and repellents. In Thailand, mosquito control and personal protection from mosquito bites are currently the most important measures for preventing and controlling mosquito-borne diseases. Although there are various control strategies for dengue disease, participation from the local community plays a vital role in the success of disease control. At present, a lot of local people have seen the value of local indigenous knowledge and used this to improve their life. The local community in the southern part of Thailand has used mosquito repellent from local knowledge for a long time. The problem regarding mosquito repellent made from local indigenous knowledge is that it has not yet been tested to determine its effectiveness. Therefore, this research aims to assess the effectiveness of mosquito repellent from local learning from Nakhon Si Thammarat provinces in Thailand. From the survey, out of 23 districts, six mosquito repellents were found in 3 communities, including Nabon, Muang, and Thasala. The repellent efficacy against the laboratory strain of *Aedes aegypti* by using the human-bait technique of the WHO (1996) standard method, with slight modifications. Approximately 0.1 ml of each test sample was applied evenly onto a 30 cm^2^ test site on one forearm of each human volunteer. Exposure experiments continued at 30 min intervals until at least two bites occurred in a 3-min period, or when a first bite was followed by a confirming bite (second bite) in the subsequent observation period. Each test was duplicated on different days for the two human volunteers. The result shows that three mosquito repellents made from local indigenous knowledge that have protection that lasts for more than 2 h are Ban Ko Sa Child Development Center's citronella spray (Nabon district, Kaew Saen subdistrict), Khun Lang's citronella spray, and Khun Lang's citronella ointment (Muang district, Pak Phun subdistrict). The result of this research was reported back to the local community to re-evaluate their self-reliance on their protection against mosquito biting.

## 1 Introduction

Interest in the control of *Aedes aegypti* lies in the fact that they act as vectors of dengue and dengue hemorrhagic fever, respectively, which are serious public health problems in Thailand and many developing countries. Mosquitoes are vectors of several infectious diseases such as dengue, malaria, yellow fever, filariasis, Japanese encephalitis, and other major health problems in tropical and subtropical countries [1]. The current death toll from all mosquito-borne diseases is estimated to be between four hundred thousand to 1 million individuals. In comparison, 216 million individuals were infected, and 445,000 died worldwide from malaria in 2016.

Various synthetic and botanical-derived chemicals have been studied to repel mosquitoes. However, few repellents are effective and safe enough to be applied repeatedly to the skin. The best-known synthetic chemical is *N, N*-diethyl-3-methyl benzamide (DEET), which is accepted as the most effective broad spectrum insect repellent component, with a long-lasting effect on mosquitoes and other biting arthropods [2]. Presently, DEET is the main active ingredient in most repellents commercially available to consumers worldwide under various brand names, as 5–100 % concentrations in different formulations are applied to the skin and clothing. However, there are some rare reports of severe reactions in people; additionally, DEET melts plastics causing spoilage of equipment, such as glasses and mobile phones, and many consumers find the odor and sensation on the skin unpleasant [3]. For these reasons, many potential users prefer natural alternatives such as those based on plant extracts, for example, citronella oil from the *Cymbopogon nardus* plant and *p*-menthane-3,8- diol (PMD) from lemon eucalyptus (*Eucalyptus maculata citriodon*), which have good repellent properties [[4], [5], [6], [7]]. The disadvantage of utilizing plant-based repellents is that many are comprised of very volatile ingredients, are ineffective over long periods, and require regular reapplication.

Many researchers have focused on using active essential oils as alternatives to chemical pesticides for mosquito prevention and control. Soonwera and Phasomkusolsil [8] assessed the repellent activities of essential oils from ylang and lemon grass mixed with natural oils against *Aedes aegypti* and *Culex quinquefasciatus*. Herbal repellents exhibit higher repellent activity than IR3535 12.5 % w/w but lower repellent activity than DEET 20 % w/w. The *Cananga odorata* oil in coconut oil shows excellent activity with 98.9 % protection from bites of *Ae. aegypti* for 88.7 ± 10.4 min. Tisgratog et al. [9] investigated the mosquito-repellent properties of 37 plant species within 14 plant families and identified five essential oils with promising insect-repellent activities. Thorsell et al. [10] tested the repellency of some natural products (*Achillea millefolium* extracts [yarrow], birch/pine tar, citronella, clove, eucalyptus, geranium, lavender, lily of the valley, and peppermint oils) in the laboratory against *Ae. aegypti* and in the field against *Ae. communis* and *Ae. cinereus*. Laboratory tests showed that yarrow extract exhibits similar repellency to DEET. Essential oils from natural herbs with low toxicity and high safety for humans are good alternative chemical repellents.

In Thailand, local people have indigenous knowledge and/or have seen the value of local wisdom and used this to improve their life. For example, in the Northeastern part of Thailand, the villagers of the Ban Don Han community learning center (Ban Fang subdistrict, Ban Fang district, Khon Kaen province) have made citronella-based repellent and spray on the bushes around the house to prevent mosquitoes from entering into their home. The problem is when the smell of citronella fades, mosquitoes will return. With the local/indigenous knowledge and determination of the villagers, it led to the transformation of citronella into a mosquito-repellent herbal mat by weaving the fibers of the citronella mixing with the papyrus woven into a mat. In another region of the country, citrus, basil, phlai, guava, eucalyptus, celery, cloves, and ylang-ylang have been used to protect their body from mosquito bites in various forms such as Chonthicha herbal incense-folk wisdom-based mosquito repellent, citrus spray, celery gel, phlai-clove lotion, etc. Moreover, Kumlert et al., 2017 [11] studied the local wisdom of mosquito-repellent made from local knowledge in the South of Thailand. From the survey, 11 mosquito-repellents were found in 7 provinces, including Krabi, Phatthalung, Phangnga, Yala, Pattani, Trang, and Narathiwat province. The result indicated that two mosquito-repellents have an average protection time lasting more than 2 h which are biore orange cream 2.10 h (Krabi) and citronella-tamaindus milk cream 4.36 h (Phatthalung). Mix-herbal boiled water, tobacco fermented water, citronella belt, citronella fermented water, giloy, potpourri, citronella-lime powder, citronella smoked shirt, and citronella soaked clothes showed less efficacy in repelling *Aedes aegypti* with the average protection time of 47, 28, 39, 1, 1, 1.4, 1, 2, and 1 min, respectively. Consequently, this study investigated the repellency of local indigenous knowledge-based repellent samples collected from Nakhon Si Thammarat province against the *Ae. aegypti* mosquito under laboratory conditions.

## 2 Materials and methods

### 2.1 Study area

**Fig. 1:**
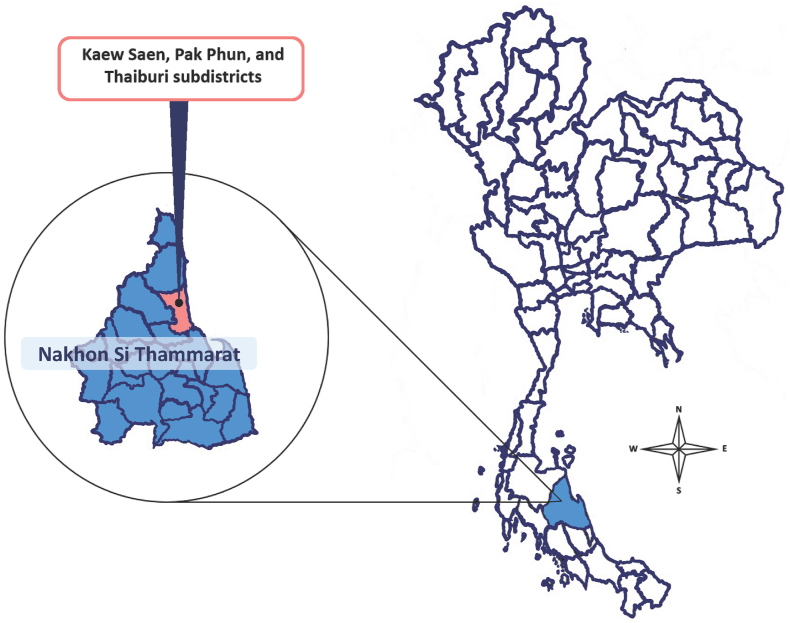
**Map of Thailand shows the collection sample sites of local indigenous knowledge-based repellent used for the investigation.** Samples were collected from three subdistricts (Kaew Saen, Pak Phun, and Thaiburi) in the Muang, Nabon, and Thasala district of Nakhon Si Thammarat province, southern Thailand.

Our preliminary survey was conducted by searching for online information and inquiries to Public health agencies, which can be contacted in the area where there are villagers in the community who have used local indigenous knowledge-based repellent in applying anti-mosquito bites. A survey of repellent made from local wisdom focused on Nakhon Si Thammarat province, the local wisdom-based repellent samples were collected from Nabon, Muang, and Thasala district, Nakhon Si Thammarat province: Kaew Saen, Pak Phun, and Thaiburi subdistrict (Fig. 1). Repellent efficacy trials were investigated after receiving permission from the possessor of this local indigenous knowledge-based repellent samples.

We focus on this region due to the information from the Office of Disease Prevention and Control 11, Department of Disease Control, Ministry of Public Health, Thailand. They reported that Muang Nakhon Si Thammarat, Na Bon, and Tha Sala district a high-risk area of dengue fever in the South of Thailand year 2022. In 2023, dengue fever has spread since the beginning of the year, from January to May 2023, and dengue fever patients reached 18,173 cases, 4.2 times more than last year. The dengue situation in Nakhon Si Thammarat province, it was found that there are many areas of concern, especially in Khanom, Muang, Cha-uat, and Tha Sala districts, with 1809 cumulative cases of dengue fever (907 males and 902 females) from January to June 2023, and have not yet been a survey of mosquito-repellent made from local indigenous knowledge in this high-risk areas.

### 2.2 Repellent samples

**Fig. 2:**
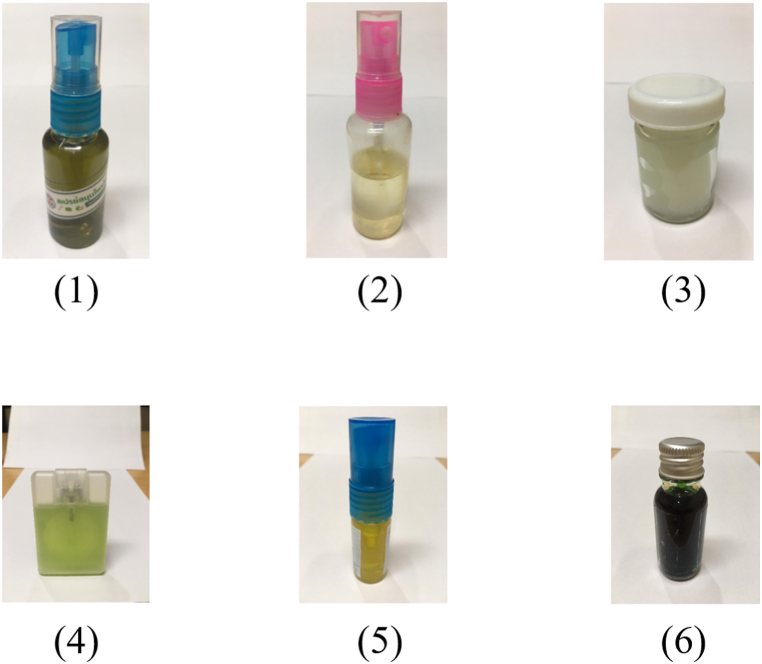
Local indigenous knowledge-based repellent samples were used in this study.

A total of 6 local indigenous knowledge-based repellent samples were collected from Nabon district, Kaew Saen subdistrict, Muang district, Pak Phun subdistrict, and Thasala district, Thaiburi subdistrict Nakhon Si Thammarat province: 1) Ban Ko Sa Child Development Center's citronella spray (Fig. 2 (1)); 2) Khun Lang's citronella spray (Fig. 2 (2)); 3) Khun Lang's citronella ointment (Fig. 2 (3)); 4) Citronella-citrus spray (Fig. 2 (4)); 5) Phlai-khamin spray (Fig. 2 (5)); and 6) Morakot's green oil (Fig. 2 (6)).

The ingredient details of 6 local indigenous knowledge-based repellent samples in brief as follows:

1) Ban Ko Sa Child Development Center's citronella spray: Citronella grass + Aqua (Fig. 2 (1))
2) Khun Lang's citronella spray: Citronella grass + Ethanol (Fig. 2 (2))
3) Khun Lang's citronella ointment: Citronella grass + Ethanol + Vaseline petroleum jelly (Fig. 2 (3))
4) Citronella-citrus spray: Kaffir lime + Citronella grass + Aqua + Camphor (Fig. 2 (4))
5) Phlai-khamin spray: Cassumunar ginger + Turmeric + Vegetable oil + Borneo camphor + Mentol + Camphor (Fig. 2 (5))
6) Morakot's green oil: Pandan + Bai-ya-nang + Gotu kola + *Clinacanthus nutans* (Burm.f) Lindau. + Aqua (Fig. 2 (6))

An example of an effective local indigenous knowledge-based repellent samples procedure is as follows:

1) Ban Ko Sa Child Development Center's citronella spray

**Figure:**
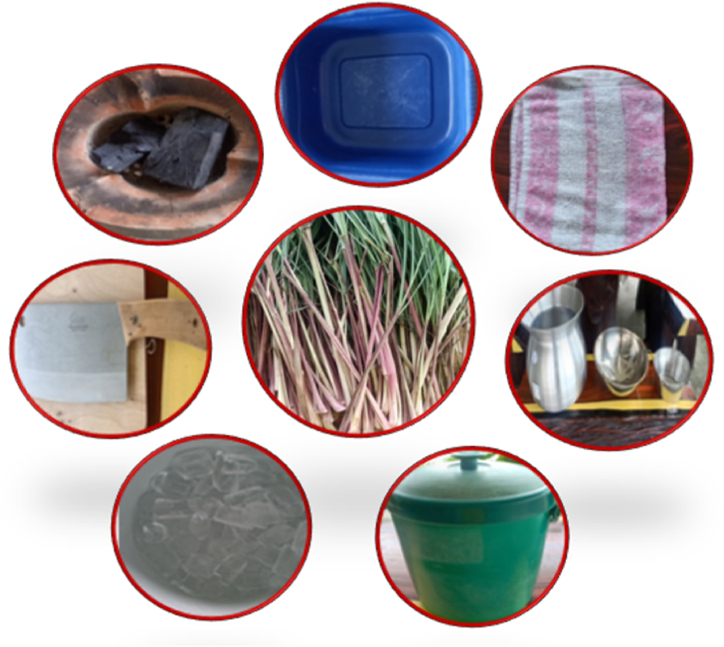

### 2.3 Materials

1. Basin
2. Cleaver
3. Chopping board
4. Brazier and charcoal
5. Ice
6. Citronella grass (stalks and leaves)
7. Steaming pot
8. Small towel
9. Vacuum flask

Steps.

1. Wash and cut off all parts used of Citronella grass, approximately 2-inch pieces.
2. Citronella grass extraction: isolation of volatile oil was achieved by water distillation of the cutoff material of Citronella grass.
  2.1 Plant material was placed in the steaming pot with water connected to a basin on top containing ice.
  2.2 The steaming pot was heated and allowed to boil until the distillation was complete.
3. The liquid formed, together with volatile oil, was collected and kept in a brown bottle.
4. Preparations of local indigenous knowledge-based repellent samples, the Citronella oil dissolved in a proper vehicle and investigated for repellent activity against *Ae. aegypti* under laboratory conditions.

**Figure:**
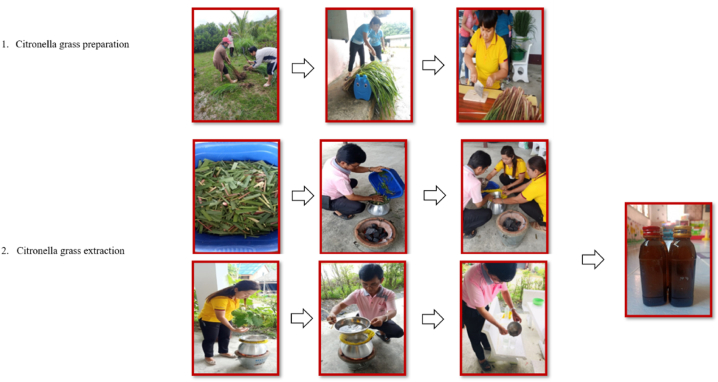

2) Khun Lang's citronella spray
  1. Wash and cut off five stalks & leaves of Citronella grass into finely ground.
  2. Put the plant samples in a glass bottle and pour the alcohol over them (300 ml).
  3. Fermented for seven days
  4. The mixtures were filtered through white cotton (stock solution)
  5. Packed in a spray bottle.
3) Khun Lang's citronella ointment
  1. The 150 ml filtered mixtures (stock solution) were mixed with 85 g of Vaseline petroleum jelly.

This project was approved and conducted according to protocol WUEC-21-263-01 of the Ethics Committee in Human Research Walailak University and WU-ACUC-64032 of the Animal Ethics Committee, Walailak University, Thailand.

### 2.4 Mosquitoes test populations and rearing

The *Ae. aegypti* used in this study were laboratory colonies originating from specimens collected in Chiang Mai province, northern Thailand (Muang Chiang Mai-susceptible: MCM-S). The colonies were housed without exposure to any pathogens or insecticides under controlled insectary conditions (25–30 °C, 80–90 % R.H., and 14:10 h light/dark photoperiod) at the Department of Parasitology, Faculty of Medicine, C.M.U. The guideline procedure of Limsuwan et al., 1987 was modified slightly for mass-rearing methods [12]. Larvae reared in plastic trays were fed on finely ground dog biscuits. Adults in screened cages were provided with continuous access to 10 % sucrose and 10 % multivitamin syrup. Five to seven days old female mosquitoes were starved before repellent testing by providing them with only distilled water for 8–12 h.

## 3 Repellent test

### 3.1 Human volunteers

Healthy human volunteers with no history of allergic reactions or dermatological disease to arthropod bites were selected for investigating repellent efficacy. All volunteers were informed of the methodology, probable discomforts to subjects, and remedial arrangements before signing an informed consent form.

### 3.2 Investigation of repellent activity

Local indigenous knowledge-based repellent samples, including Ban Ko Sa Child Development Center's citronella spray, Khun Lang's citronella spray, Khun Lang's citronella ointment, Citronella-citrus spray, Phlai-khamin spray, and Morakot's green oil were screened for repellent efficacy against the MCM-S laboratory strain of *Ae. aegypti* using the human-bait technique of the WHO (1996) standard method, with slight modifications. Repellency determinations were performed in a 10 × 10 × 3 m room at room temperature 27–30 °C and relative humidity of 67–80 %. The testing period was conducted between 0800 and 1600 h. Two hundred and fifty unfed female mosquitoes were selected at random and placed inside a standard mosquito cage (30 × 30 × 30 cm), which provides a density resting surface (or vertical resting surface per mosquito) of 14.4 [13]. Before the application of the test samples, the arms of each volunteer were washed with water, and a plastic sleeve covered the ventral part of the forearm with a rectangular portion cut out (3 × 10 cm), thus exposing the treated area only. A rubber glove protected the hand. Approximately 0.1 ml of each sample was applied smoothly onto a 30 cm^2^ test area on one forearm of each volunteer. The other forearm acted as a control and was treated with absolute ethanol by the same protocol as the test repellent. After air drying for 1 min, the control arm was put into a mosquito cage for 3 min to check mosquito bite activity. If at least two mosquitoes landed on the control arm, the repellency test was performed by similarly exposing the treated forearm. The control and test arms were switched alternately to test the readiness of the mosquitoes to bite and prevent any bias. The complete protection time was recorded after exposing the treated forearm for 3 min at 30-min intervals until either two bites occur in a single exposure period or one bite occurs in each of two consecutive exposure periods. After each experiment, the tested mosquitoes were discarded. Each test was duplicated on different days for the two human volunteers. No one tested more than 1 sample per day. Randomization was used to assign the order of tests and treatment of volunteers blinded to the repellent applied.

### 3.3 Satisfaction survey

The questionnaire used in the survey was used to measure overall satisfaction, safety satisfaction, advice products to other people, price, and cost-effective satisfaction by modifying the customer satisfaction in the sore throat medicine population in Bangkok and perimeter areas [14]. The 324 people who consumed the effective 1 of the three local indigenous knowledge-based repellent samples to prevent mosquito bites in the past three months were selected as the sample group. Three experts assessed the content validity of the questionnaire in parasitology or related fields. Thirty individuals pretested the reliability of the questionnaire in a nonstudy sample population who lived outside the study area, and Cronbach's alpha coefficient for the whole questionnaire was calculated to be 0.76. The Content Validity index (CVI) for the constructs of the questionnaire was 0.9.

### 3.4 Chemical analysis of mosquito repellent

The chemical profiles of Khun Lang's citronella spray (the most effective sample) and Citronella-citrus spray (the non-effective sample) were determined by gas chromatography/mass spectrometry (GC/MS) analysis at the Science and Technology Service Center, CMU. This GC-MS system consisted of Hewlett-Packard GC 7890A Agilent Technology interfaced to a single quadrupole mass selective detector, MSD 5975C (EI) Agilent Technology. The column was a DB-5MS (30 m × 0.25 mmID × 0.25 μm film thickness). The total GC/MS running time was 60 min with the operating conditions programmed as follows: the oven temperature was 50–300 °C at 5 °C/min. The carrier gas was helium at a constant flow rate of 1.0 ml/min; the injector port and detector temperature were 250 °C and 280 °C, respectively. The diluted sample (0.1 μl) was injected by splitting, with a split ratio of 50:1. Mass spectra were obtained at 70 eV and recorded in the 50–550 amu. The interface and iron trap temperature was 230 °C and 150 °C, respectively. The chemical constituents of each plant extract were identified by their retention time and computer matching with the Wiley 8N08 spectral library and comparing their mass spectra with those of authentic samples. The relative percentage amount of each component was calculated by comparing its average peak area to the total area.

## 4 Results

### 4.1 Demographic characteristics

The data profile collected from 324 subjects in this study is given in Table 1. They comprised those aged 20 and above; among them, 126 (38.9 %) were men, and 198 (61.1 %) were women. The mean age of the subjects was 40.8 ± 19.5 years. A quarter of the respondents (25.5 %) had basic primary-level education. Only 32 (15.7 %) of the respondents were formally employed. The majority (65.20 %) of respondents were married. Overall, 36.80 % (75/324) of the respondents had an income of 5001–10,000 baht/month (142.34–284.62 USD). In 2022, the income per capita of Thailand is estimated at 244,838 baht (6968.48 USD) per person per year. It can be regarded that the respondents' pay is lower than the income per capita of Thailand.

**Table 1:** The Demographic characteristics of the sample (n = 324).

Characteristics		Number	Percent (%)
Sex			
Male		74	36.30
Female		130	63.70
Age (years)			
20-30		58	28.40
31-40		64	31.40
41-50		37	18.10
51-60		22	10.80
> 60		23	11.30
Marital status			
Single		49	24.00
Married		133	65.20
Divorced/Widowed		22	10.80
Education			
Primary School		52	25.50
Secondary and high school diploma		53	26.00
Associate degree		43	21.10
Bachelor degree		50	24.50
Master degree		6	2.90
Occupation			
Student		14	6.90
Government employee/State enterprise employee		32	15.70
Private employee		31	15.20
Agriculturalist		44	21.60
Business owners		35	17.20
Fishery		8	3.90
Housekeeper		11	5.40
General contractor		3	1.50
Others		26	12.70
The income per month, Baht			
< 5000		26	12.70
5001–10,000		75	36.80
10,001–15,000		46	22.50
15,001–20,000		22	10.80
> 20,000)		35	17.20

### 4.2 Repellent test

The topical application of six local indigenous knowledge-based repellent samples effectively protected against mosquitoes, with varying degrees of repellency. Only three local indigenous knowledge-based repellent samples, including Khun Lang's citronella spray, Ban Ko Sa Child Development Center's citronella spray, and Khun Lang's citronella ointment, produced impressive results repellent activity against *Ae. aegypti* with median complete-protection times of 4.25, 3.25, and 2.50 h, respectively (Table 2). The other three samples (Citronella-citrus spray, Phlai-khamin spray, and Morakot's green oil) appeared ineffective. No local skin reaction such as rash, swelling, irritation, or other allergic responses was observed during the study period.

**Table 2:** Repellent activity of local indigenous knowledge-based repellent samples against female *Ae. aegypti*.

Local indigenous knowledge-based repellent samples	Median complete-protection time (Range, h)^a^
Ban Ko Sa Child Development Center's citronella spray	2.50 (2.0–3.0)
Khun Lang's citronella spray	4.25 (4.0–4.5)
Khun Lang's citronella ointment	3.25 (3.0–3.5)
Citronella-citrus spray	0.00 (0.0–0.0)
Phlai- khamin spray	0.00 (0.0–0.0)
Morakot's green oil	0.00 (0.0–0.0)
**A positive control (DEET)**	6.25 (5.0–6.5)

a There were 4 replicates of each test.

### 4.3 Satisfaction survey

The satisfaction was collected using a questionnaire that showed 18 items in 3 parts: part 1: personal information (Table 1), part 2: local wisdom-based repellent products information, and part 3: satisfaction in choosing local wisdom-based repellent products to repel mosquitoes (Table 3). The statistics used to analyze data was percentage. The product usage data (part 2) and satisfaction (part 3) are shown in Table 3, Table 4, respectively.

**Table 3:** Product usage data.

Product usage data	Number	Percent (%)
Ever used local indigenous knowledge-based repellent products form		
Spray	301	92.9
Ointment	23	7.1
Frequency use form		
Spray	303	93.5
Ointment	64	31.4
Product factors		
Efficacy	274	84.6
Easy to use	276	85.2
Prize	254	78.4
Odor	277	85.5
Packaging	229	70.7
Safety	236	72.8
Frequency use per week		
Everyday	178	54.9
4–5 times	46	14.2
2–3 times	76	23.5
One time	23	7.1
Expected protection time (h.)		
1-5	81	25.0
6-10	6	1.90
11-15	4	1.20
16-24	217	67.0

**Table 4:** Satisfactions of local indigenous knowledge-based repellent products used.

Satisfactions of local indigenous knowledge-based repellent products used	Number	Percent (%)
Local indigenous knowledge-based repellent products can repel mosquitoes		
Very good	71	21.9
Good	183	56.5
Moderate	64	19.8
Low	0	0.0
Very low	6	1.9
Local indigenous knowledge-based repellent products have standard quality		
Very good	111	34.3
Good	146	45.1
Moderate	61	18.8
Low	0	0.0
Very low	6	1.9
Satisfaction of applied local indigenous knowledge-based repellent products on the skin		
Very good	112	34.6
Good	156	48.1
Moderate	50	15.4
Low	0	0.0
Very low	6	1.9
Local indigenous knowledge-based repellent products are better than products manufactured in the market		
Very good	113	34.9
Good	135	41.7
Moderate	70	21.6
Low	0	0.0
Very low	6	1.9
I will recommend others to use the local indigenous knowledge-based repellent products to repel mosquitoes		
Very good	114	35.2
Good	158	48.8
Moderate	46	14.2
Low	0	0.0
Very low	6	1.9
The local indigenous knowledge-based repellent products safe to applied on the skin to repel mosquitoes		
Very good	129	39.8
Good	157	48.5
Moderate	31	9.6
Low	1	0.3
Very low	6	1.9

From Table 3 and it was found that most of the respondents had used local indigenous knowledge-based repellent products such as mosquito repellent sprays (92.9 %) rather than ointments, and the most often used form was the spray (93.5 %), followed by the formation of ointment cream (6.5 %). The main factor or reason that users choose to use local indigenous knowledge-based repellent products is to repel mosquitoes, followed by fragrant, easy to use & easy to carry, and a long protection time with 85.5 %, 85.2 %, and 84.6 %, respectively. The respondents used local indigenous knowledge-based repellent products daily (54.9 %), followed by 2–3 times a week (23.5 %), with the expected protection time against mosquito bites in the range of 16–24 h (67.0 %).

From Table 4 and it was found that the respondents thought that the local indigenous knowledge-based repellent products could repel mosquitoes with a good level of 56.5 %. The quality of local indigenous knowledge-based repellent products is at a good level of 45.1 %. In addition, most respondents were satisfied with local indigenous knowledge-based repellent products, at a good level of 48.1 %. The respondents thought that local indigenous knowledge-based repellent products could repel mosquitoes like commercial products, at a good level of 41.7 %. Most respondents need to recommend others to use these products good level of 48.8 %. It's pretty safe to apply on the skin at a good level of 48.5 %, and the overall satisfaction with local indigenous knowledge-based repellent products is 48.5 %.

### 4.4 Chemical analysis of mosquito repellent

In this study, GC-MS was the technique chosen for chemical analysis. It was used to show the profile of constituents and characterize the main active substances that were possibly responsible for the most effective repellent potential of local indigenous knowledge-based repellent products. GC/MS analysis of Khun Lang's citronella spray products led to the identification of 30 different constituent compounds, representing 99.99 % of the total content (Table 5). The ineffective sample: Citronella-citrus spray showed 31 compounds in Table 6.

**Table 5:** Chemical constituents of Khun Lang's citronella mosquito repellent spray.

Chemical constituent	Retention time	%Composition
Pinene	6.128	0.358
1,4-Cineol	8.157	0.015
*o*-Cymene	8.413	0.020
⟨-Limonene	8.543	0.092
1,8-Cineol	8.636	0.015
(+-)-Linalool	10.461	0.014
Fenchol	11.046	0.015
1-Methyl-4-(1-methylethyl)-3-cyclohexen-1-ol	11.501	0.024
(−)-L-isopulegol	11.874	0.078
®-Citronella	11.940	0.322
(−)-Isopulegol	12.167	0.109
1,7,7-Trimethylbicyclo[2.2.1]heptan-2-ol	12.544	0.010
Salicylic acid	13.557	96.011
®-Citronellol	14.055	0.288
trans-Geraniol	14.693	0.379
cis-Citral/Neral	15.164	0.010
Benzoic acid,2-methoxy-, methyl ester	16.898	0.018
Terpin	17.084	0.011
2,6-Octadiene,2,6-dimethyl	17.344	0.070
Phenol,2-methoxy-4-(2-propenyl)	17.397	0.006
Geraniol acetate	18.088	0.070
(−)- ®-Elemene	18.461	0.054
(+)-Longofolene	19.018	0.021
4-Hydroxybenzoic acid methyl ester	19.958	0.053
⟨-Amorphene	20.619	0.011
⟨-Muurolene	21.197	0.023
©-Cadinene	21.561	0.021
(+)-*D*-Cadinene	21.673	0.054
Elemol	22.381	0.073
Unknown	23.788	1.627
Unknown	24.652	0.090
(−)-T-Muurolol	24.879	0.036
**Total identified**		**99.998**
**No. of identified constituents**		**30**

**Table 6:** Chemical constituents of Citronella citrus mosquito repellent spray.

Chemical constituent	Retention time	%Composition
1R-⟨-Pinene	5.885	0.424
2, 2-Dimethyl-3-methylenenorbornane	6.285	0.056
®-Pinene	7.002	4.191
®-Myrcene	7.207	0.142
*p*-Cymene	8.178	0.449
(+-)-⟨-Limonene	8.330	7.786
©-Terpinene	9.067	1.250
Unknown	9.426	0.091
3-Carene	10.205	0.073
Unknown	10.647	0.332
(+)-Fenchol	10.786	1.074
⟨-Fenchene	10.916	0.040
Alcanfor	11.577	2.697
©-Terpinen	11.896	0.106
Unknown	12.189	35.182
Borneol	12.490	40.524
1-Isopropyl-4-methyl-1,4-cyclohexadiene	12.586	2.138
R(+)-Limonen	12.929	0.181
Bicyclo[2.2.1]heptane, 2,2-dimethyl-3-methylene	13.008	0.278
Unknown	13.422	1.235
Unknown	13.739	0.062
1,4-Cyclohexadiene, 1-methyl-4-(1-methylethyl)	13.838	0.091
3-Carene	14.381	0.101
Unknown	14.530	0.106
Unknown	15.477	0.131
(−)-⟨-Copaene	17.797	0.643
Unknown	18.035	0.088
Unknown	18.451	0.046
®-Caryophyllene	18.933	0.238
(+)-δ-Cadinene	21.351	0.139
**Total identified**		**99.894**
**No. of identified constituents**		**31**

**Fig. 3:**
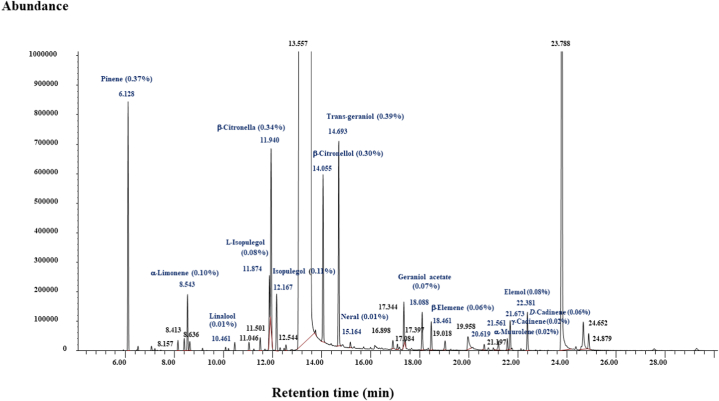
The chromatogram obtained by GC/MS analysis of Khun Lang's citronella mosquito repellent spray.

**Fig. 4:**
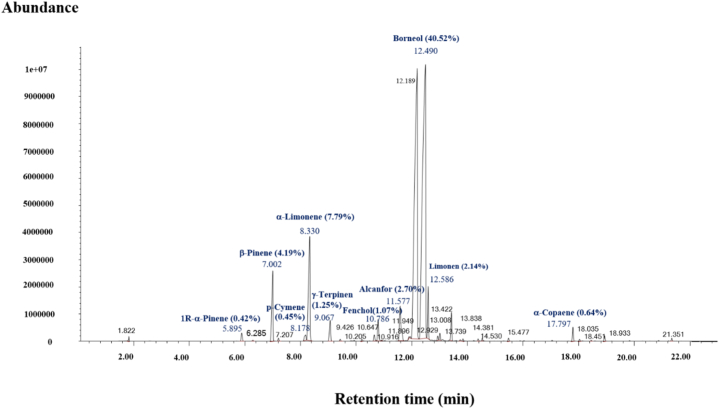
The chromatogram obtained by GC/MS analysis of Citronella citrus mosquito repellent spray.

It was found that the compositions in these Khun Lang's citronella spray were almost similar as citronella, citronellol, geraniol, and Pinene were principal constituents showing the highest peaks (Fig. 3). Thirty chemical components were identified during the phase (Table 5). They are Pinene (0.36 %), 1,4-Cineol (0.02 %), *o*-Cymene (0.02 %), ⟨-Limonene (0.9 %), 1,8-Cineol (0.02 %), (+-)-Linalool (0.01 %), Fenchol (0.02 %), 1-Methyl-4-(1-methylethyl)-3-cyclohexen-1-ol (0.02 %), (−)-L-isopulegol (0.08 %), ®-Citronella (0.32 %), (−)-Isopulegol (0.11 %), 1,7,7-Trimethylbicyclo[2.2.1]heptan-2-ol (0.01 %), Salicylic acid (96.01 %), ®-Citronellol (0.29 %), trans-Geraniol (0.38 %), cis-Citral/Neral (0.01 %), Benzoic acid,2-methoxy-,methyl ester (0.02 %), Terpin (0.01 %), 2,6-Octadiene,2,6-dimethyl (0.07 %), Phenol,2-methoxy-4-(2-propenyl) (0.01 %), Geraniol acetate (0.07 %), (−)-®-Elemene (0.05 %), (+)-Longofolene (0.02 %), 4-Hydroxybenzoic acid methyl ester (0.05 %), ⟨-Amorphene (0.01 %), ⟨-Muurolene (0.02 %), ©-Cadinene (0.02 %), (+)-*D*-Cadinene (0.05 %), Elemol (0.07 %), and (−)-T-Muurolol (0.04 %). The most abundant compound was Salicylic acid (96.01 %), whereas *trans*-geraniol (0.39 %), Pinene (0.37 %), ®-Citronella (0.34 %), ®-Citronellol (0.30 %), Isopulegol (0.11 %), ⟨-Limonene (0.10 %), and L-isopulegol (0.08 %) were minor constituents (Fig. 3). The main chemical component of non-effective (Citronella-citrus) spray was Borneol (96.01 %), followed by ⟨-Limonene, ®-Pinene, Alcanfor, and ©-Terpinene (Fig. 4).

The chemical compositions, with citronellal, citronellol, and geraniol predominating, of Khun Lang's citronella mosquito repellent spray samples, are quite compatible; this may be due to the similar extraction techniques from the same plant species, harvested from the same place in Nakhon Si Thammarat province.

## 5 Discussion

From 6 local indigenous knowledge-based repellent samples, only three herb materials, provided impressive results of repellent activity against *Ae. aegypti* with median complete-protection times of 4.25, 3.25, and 2.50 h. The initial success of this study is the protection time of up the 2.0 h from three herbal products, including Khun Lang's citronella spray, Ban Ko Sa Child Development Center's citronella spray, and Khun Lang's citronella ointment. This meets the requirement of the Food and Drug Administration (FDA), which needs a minimum protection time of 2 h before allowing sales of repellents in Thailand. Furthermore, no local skin reaction such as rash, swelling, irritation, or other allergic responses was observed during the study period.

In the preliminary survey data, we found more local indigenous knowledge-based repellents than six in Nakhon Si Thammarat province and nearby; unfortunately, we collected only six samples which are gradually disappearing from the area due to Covid 19 pandemic and the duration and budget constraints, it may still have local indigenous knowledge that has not yet been discovered in this study. Furthermore, the ingredients of each local indigenous knowledge-based repellent formula and the protection time compared with DEET (a standard insecticide) were reported briefly.

Most of the respondents were satisfied with local indigenous knowledge-based repellent in the form of repellent spray with the reasons of good smell, easy to use and carry, which is not consistent with the study of Mitpratan [15] that the most popular feature of sun protection body lotion was lotion and then cream. Weerapreeyakul et al. [16] study of satisfaction with applying black sesame meal scrub cream found that the volunteers were satisfied with the texture, odor, and viscosity. Hence further development of the highest protection time of local indigenous knowledge-based repellent needs to be conducted as and stability test. To make the community-based products more attractive to the users, they should be improved, such as the appearance, odor, and local herbal wisdom efficacy information. The users have a high level of satisfaction with the quality and safety of herbal wisdom products, which gives them confidence in the creation and encourages them to continue using the product, ultimately resulting in them recommending the product to others. This is accordant with Suthprasertporn et al. study of consumer satisfaction in using throat relief products in Bangkok [14]. The results concluded that customers would be loyal to the product and repeat use if they were delighted with the outcome. The study of Suksomnirundorn found that when customers are satisfied with the product, it leads to customer loyalty. Repeated product purchases influence building trust with new customers [17].

The composition of Khun Lang's citronella spray products in this study was slightly different from that previously reported by Maia and Moore; *Cymbopogon nardus* or citronella grass contains the components of citronellal, citronellol, geraniol, citral, α Pinene, and limonene [18]. The results of Nakahara et al. indicated a total of 16 compounds were identified from GC–MS analysis of *C. nardus* essential oil (CN oil) and the main compounds were citronellal (27.53 %), citronellol (25 %), and nerol (21.89 %) [19]. The capillary GC and GC-MS analyses of CN oil indicated that the major constituents were monoterpenes: geraniol (35.7 %), *trans*-citral (22.7 %), *cis*-citral (14.2 %), geranyl acetate (9.7 %), citronellal (5.8 %) and citronellol (4.6 %) predominated in the oil. The most active compounds among the 16 examined volatiles, consisting of 6 major constituents of the essential oil and 10 other related monoterpenes were citronellal and linalool. Saputra et al. [20], indicated CN oil predominant compounds presence of ammonium carbamate (18.26 %), carbinol (13.57 %), neophytadiene (11.65 %), *trans*-geraniol (6.92 %), phenol-methoxy (6.15 %), norolean (4.93 %), benzofuran (3.9 %), guaiacol (3.23 %), hexadecen-phytol (3.1 %), beta-citronellol (2.69 %), *trans*-caryophyllene (2.61 %), alphahumulene (2.45 %), and valerol (2.38 %) quantified by GCMS Shimadzu QP2010. GC/MS analysis of *Cymbopogon nardus* essential oil reported by Kaur et al.; showed the presence of citronellal (22.15 %), citronellol (16.34 %), and geraniol (11.16 %) as major constituents [21]. The composition of the CN oil from *C. nardus* cultivated in India was significantly different from this study. The major components were citronellal (29.7 %), geraniol (24.2 %), γ-terpineol (9.2 %), and *cis*-sabinene hydrate (3.8 %) [22].

It is generally recognized that there are several chemical phenotypes (chemotypes) compounds in the same plant, such as categories of cannabis [23]. It was reported that plant samples in most classification studies were collected from dissimilarity origins [24] and are subject to inconsistent environmental factors during the growth phases and post-harvest treatment [25,26]. Furthermore, improper plant sample preparation, extraction procedures, and isolation techniques during laboratory investigation may influence classification results [27]. Total factors provide to the variation in chemical profiles of the plant products, which in turn leads to inconsistent results and poor classification accuracy. Increased precise classification results are acceptable when plants are grown in a sole location, under undifferentiated environmental conditions, including the cultivation conditions of the plants, and evenly processed [28].

The antibacterial [29,30], antihelmintic [31], antiparasitic [32], anti-inflammatory [33], anticonvulsant [34], and antioxidant effects [[35], [36], [37]] of CN oil and its constituents are well-known as plant-based insect repellents. These can be used as traditional insect repellents with minimal adverse side effects [38]. The CN oil extracted from *C. nardus* was shown to be a practical mosquito repellent on human subjects for up to 2 h [38]. The exhibited protection of CN oil against biting from three mosquito species: *An. minimus*, *Cu. quinquefasciatus*, and *Ae. aegypti*, were 130 min, 140 min, and 115 min when using 0.9 %, 0.8 %, and 0.8 %, respectively. The protection time in minutes and percentage of Thai CN oil protection against *Ae. aegypti* was 58.33 min and 96.92 % at 0.17 μl/cm^2^, respectively [39]. In the study of Sutthanont et al. [40], they reported the repellent efficacy of CN oil (a positive control) compared to the repellent activity of 10 undiluted essential oils (anise, basil, bergamot, coriander, patchouli, peppermint, petitgrain, rosemary, sage, and vetiver) against *Ae. aegypti*, *An. dirus*, and *Cu. quinquefasciatus*. Petitgrain oil was the most effective against *Ae. aegypti* (270 min), whereas CN oil showed complete-protection time from bites of *Ae. aegypti* for 180 min. Sritabutra and Soonwera demonstrated the protection time of citronella grass oil in olive oil & coconut oil against Ae. aegypti and Cu. quinquefasciatus was 54 and 165 (min) & 82 and 105 (min), respectively [41]. Tjahjani found the same result and reported that the citronella oil was effective against Aedes a*nd Culex* species (287 and 40 min protection time, respectively) [42]. In comparison, we did not find the potent component with the mosquito-repelling properties of non-effective local indigenous knowledge-based repellent. It may be that the local repellent formulation has a minor amount of CN oil in the mixture ingredients caused undetected by GC-MS.

In conclusion, the findings of this study demonstrate the period of protection time against *Ae. aegypti* for three local indigenous knowledge repellent candidates tested reached the Food and Drug Administration (FDA) requirements for sale in Thailand at a determined time of greater than 2 h. Nevertheless, some local indigenous knowledge samples are not as practical against mosquito vectors as cited properties. This may be due to the components of the mosquito repellent in the local indigenous knowledge that is used; the amount of substance or active constituent is not enough, so it did not show any repellent or fragile activity.

## Author contribution statement

All authors listed have significantly contributed to the development and the writing of this article.

## Funding

School of Public Health, 10.13039/501100010034Walailak University PH-Prelim 2022/02 and EC for DACH-Prelim 2022.

## Data availability statement

The data that has been used is confidential.

## Additional information

No additional information is available pertaining to this article.

## Declaration of competing interest

The authors declare the following financial interests/personal relationships which may be considered as potential competing interests:Danita Champakaew reports financial support, article publishing charges, and equipment, drugs, or supplies were provided by 10.13039/501100010034Walailak University, Thailand and Chiang Mai University, Thailand.

## References

[1] Service M.W. (1983). Biological control of mosquitoes- has it a future?. Mosq. news.

[2] Thongsripong P., Green A., Kittayapong P., Kapan D., Wilcox B., Bennett S. (2013). Mosquito vector diversity across habitats in central Thailand endemic for dengue and other arthropod-borne diseases. PLoS Neglected Trop. Dis..

[3] Trongtokit Y., Rongsriyam Y., Komalamisra N., Apiwathnasorn C. (2005). Comparative repellency of 38 essential oils against mosquito bites. Phytother Res..

[4] Curtis C.F., Lines J.D., Ijumba J., Callaghan A., Hill N., Karimzad M.A. (1987). The relative efficacy of repellents against mosquito vectors of disease. Med. Vet. Entomol..

[5] Trigg J.K., Hill N. (1996). Laboratory evaluation of a eucalyptus-based repellent against four biting arthropods. Phytother Res..

[6] Trigg J.K. (1996). Evaluation of a eucalyptus-based repellent against *Anopheles* spp in Tanzania. J. Am. Mosquito. Contr..

[7] Phasomkusolsil S., Soonwera M. (2012). The effects of herbal essential oils on the oviposition-deterrent and ovicidal activities of *Aedes aegypti* (Linn.), *Anopheles dirus* (Peyton and Harrison) and *Culex quinquefasciatus* (Say). Trop. Biomed..

[8] Phasomkusolsil S., Soonwera M. (2011). Efficacy of herbal essential oils as insecticide against *Aedes aegypti* (linn.), *Culex quinquefasciatus* (say) and *Anopheles dirus* (peyton and harrison). Southeast Asian J. Trop. Med. Publ. Health.

[9] Tisgratog R., Sanguanpong U., Grieco J.P., Ngoen-Kluan R., Chareonviriyaphap T. (2016). Plants traditionally used as mosquito repellents and the implication for their use in vector control. Acta Trop..

[10] Thorsell W., Mikiver A., Malander I., Tunón H. (1998). Efficacy of plant extracts and oils as mosquito repellents. Phytomedicine.

[11] Kumlert R., Moonmek S., Sriplong W., Kolaeh K., Tawatsin A., Thavara U. (2017). Study on efficacy of local wisdom of traditional mosquito repellent to *Aedes aegypti* mosquito under community participation in the South of Thailand. Disease Control Journal.

[12] Limsuwan S., Rongsriyam Y., Kerdpibule V., Apiwathnasorn C., Chiang G.L., Cheong W.H., Sucharit S., Supavej S. (1987). Practical Entomology. Malaria and Filariasis.

[13] Gerberg E.J., Barnard D.R., Ward R.A. (1994).

[14] Sutprasertporn S. (2015). http://ethesisarchive.library.tu.ac.th/thesis/2015/TU_2015_5702030221_3583_1933.pdf?fbclid=IwAR0QPFb989GGNPcTTOnvD8cWvu9acqm0McLDmJ3lj_MXCmK5nddlLHHfjQI.

[15] Mitpratan N. (2011). Master of Business Administration.

[16] Weerapreeyakul N., Chansri N., Lawong A., Waithong S. (2013). Natural Beauty and Health through Aesthetic Science (1^st^ Decade of Aesthetic Sciences and Health and 50th Anniversary of K.K.U.).

[17] Thirawarapan S. (2015). http://pharmacy.mahidol.ac.th/th/knowledge/article/299/.

[18] Maia M.F., Moore S.J. (2011). Plant-based insect repellents: a review of their efficacy, development and testing. Malar. J..

[19] Nakahra K., Alzoreky N.S., Yoshihashi T., Nguyen H.T.T., Trakoontivakorn G. (2003). Chemical composition and antifungal activity of essential oil from *Cymbopogon nardus* (Citronella Grass). JARQ (Jpn. Agric. Res. Q.).

[20] Saputra N.A., Wibisono H.S., Darmawan S., Pari G. (2020). I.O.P. Conf. Series: Earth and Environmental Science.

[21] Kaur H., Bhardwaj U., Kaur R., Kaur H. (2021). Chemical composition and antifungal potential of Citronella (*Cymbopogon nardus*) leaves essential oil and its major compounds. J. Essent. Oil-Bear. Plants..

[22] Sritabutra D., Soonwera M. (2013). Repellent activity of herbal essential oils against Aedes aegypti (Linn.) and Culex quinquefasciatus (Say.). Asian. Pac. J. Trop. Dis..

[23] Olaizola O.A., Soydaner U., Öztürk E., Schibano D., Simsir Y., Navarro P. (2016). Evolution of the cannabinoid and terpene content during the growth of *Cannabis sativa* plants from different chemotypes. J. Nat. Prod..

[24] Kakaraparthi P.S., Srinivas K.V.N.S., Kumar J.K., Kumar A.N., Rajput D.K., Sarma V.U.M. (2014). Variation in the essential oil content and composition of Citronella (Cymbopogon winterianus Jowitt.) in relation to time of harvest and weather conditions. Ind. Crops Prod..

[25] Xavier J.K.A.M., Baia T.G.C., Alegria O.V.C., Figueiredo P.L.B., Carneiro A.R., Moreira E.C. de O. (2022). Essential oil chemotypes and genetic variability of *Cinnamomum verum* leaf samples commercialized and cultivated in the Amazon. Molecules.

[26] Perrino E.V., Valerio F., Jallali S., Trani A., Mezzapesa G.N. (2021). Ecological and biological properties of *Satureja cuneifolia* and *Thymus spinulosus* Ten.: two wild officinal species of conservation concern in Apulia (Italy). A preliminary survey. Plants.

[27] Chanthai S., Prachakoll S., Ruangviriyachai C., Luthria D.L. (2012). Influence of extraction methodologies on the analysis of five major volatile aromatic compounds of Citronella grass (Cymbopogon nardus) and lemongrass (Cymbopogon citratus) grown in Thailand. J. Assoc. Anal. Communities Int..

[28] Yasmine F.M., Mouchira A.C., Meselhy R.M., El-Sayeda A.E.K. (2020). Essential oil of Cymbopogon citratus cultivated in Egypt: seasonal variation in chemical composition and anticholinesterase activity. Nat. Prod. Res..

[29] Pattnaik V.R., Subramayam C.R., Kole S.S. (1995). Antimicrobial activity of oils from Cymbopogon inter and intraspecific differences. Microbios.

[30] De Billerbeck V.G., Roques C.G., Bessiere J.M., Fonvieille J.L., Dargent R. (2001). Effects of Cymbopogon nardus (L.) Watson essential oil on the growth and morphogenesis of Aspergillus Niger. Can. J. Microbiol..

[31] Nirmal S.A., Girme A.S., Bhalke R.D. (2007). Major constituents and antihelmintic activity of volatile oils from leaves and flowers of Cymbopogon martini Roxb. Nat. Prod. Res..

[32] George D.R., Sparagano O.A.E., Port G., Okello E., Shiel R.S., Guy J.H. (2010). Environmental interactions with the toxicity of plant essential oils to the poultry red mite Dermanyssus gallinae. Med. Vet. Entomol..

[33] Francisco V., Figueirinha A., Neves B.M., Lopes M.C., Cruz M.T., Batista M.T. (2011). Cymbopogon citratus as source of new and safe anti-inflammatory drugs: bio-guided assay using lipopolysaccharide-stimulated macrophages. J. Ethnopharmacol..

[34] Silva R.M., Ximenes R.M., Martins da C.J.G., Kalyne L., Leal A.M., Lopes de A. (2010). Comparative anticonsulvant activities of the essential oils (Eos) from Cymbopogon winterianus Jowitt and Cymbopogon citratus (D.C.) Stapf. in mice. Naunyn-Schmiedeberg’s Arch. Pharmacol..

[35] Ruberto G., Baratta M.T. (2000). Antioxidant activity of selected essential oil components in two lipid model systems. Food Chem..

[36] Hierro I., Valero A., Perez P., Gonzales P., Cabo M.M., Montilla M.P. (2004). Action of different monoterpenic compounds against Anisakis simplex S.I.L3 larvae. Phytomedicine.

[37] Khunkitti W. (2010). Essential Oil-Bearing Grasses: the Genus Cymbopogon.

[38] Moore S.J., Hill N., Ruiz C., Cameron M.M. (2007). Field evaluation of traditionally used plant-based insect repellents and fumigants against the malaria vector *Anopheles darlingi* in Riberalta, Bolivian Amazon. J. Med. Entomol..

[39] Soonwera M., Phasomkusolsil S. (2014). Efficacy of Thai herbal essential oils as green repellent against mosquito vectors. Acta Trop..

[40] Sutthanont N., Sudsawang M., Phanpoowong T., Sriwichai P., Ruangsittichai J., Rotejanaprasert C. (2022). Effectiveness of herbal essential oils as single and combined repellents against *Aedes aegypti, Anopheles dirus* and *Culex quinquefasciatus* (Diptera: Culicidae). Insects.

[41] Sritabutra D., Soonwera M. (2013). Repellent activity of herbal essential oils against Aedes aegypti (Linn.) and Culex quinquefasciatus (Say.). Asian. Pac. J. Trop. Dis..

[42] Tjahjani S. (2008). Efficacy of several essential oils as *Culex* and *Aedes* repellents. Proc ASEAN Congr Trop Med Parasitol.

